# Respecting Learner Autonomy in POCUS Image Acquisition - a Stepped Approach

**DOI:** 10.24908/pocusj.v10i02.19791

**Published:** 2025-11-17

**Authors:** David Purkarthofer, Simon Orlob

**Affiliations:** 1 Medical University of Graz, Neue Stiftingtalstraße 6, Graz, 8010, Styria, Austria; 2 Medizinercorps Graz, Austrian Red Cross, Munzgrabenstraße 151, Graz, 8010, Styria, Austria; 3 Department of Health Studies, FH Joanneum University of Applied Sciences, Alte Poststraße 149, Graz, 8020, Styria, Austria; 4 Institute for Emergency Medicine, University Hospital Schleswig-Holstein, Arnold-Heller-Straße 3, Haus 808, Kiel, 24105, Schlewig-Holstein, Germany; 5 Department of Anesthesiology and Intensive Care Medicine, Medical University of Graz, Auenbruggerplatz 5, Graz, 8036, Styria, Austria

**Keywords:** Sonography education, Point-of-care ultrasound education, Learner autonomy, Psychomotor skill development, Point-of-Care Ultrasound, POCUS

Dear Editor,

Point of care ultrasound (POCUS) is increasingly integrated into undergraduate and postgraduate medical education. While theoretical knowledge can be efficiently delivered through flipped classrooms or online modules, image acquisition remains a psychomotor skill that requires hands-on practice in small groups with a low learner-to-instructor ratio [1,2].

Instructors may be under pressure to save time or help learners to get the “right” image. In these cases, they often take the probe and manipulate it themselves. While this approach may produce an adequate sonogram in less time, it can inadvertently bypass the learner's opportunity to develop essential visuospatial understanding, problem-solving strategies, and fine motor coordination. The result is a focus on the product—a clear image—rather than the process of skill acquisition.

In our undergraduate POCUS courses, we frequently observed learners struggling with probe handling, topographic orientation, and image interpretation. These challenges were compounded by inconsistent terminology and limited hands-on learning time. To address this, we developed and implemented a graded escalation strategy designed to maximize learner autonomy, while still providing timely, structured support.

## The Four-Step Escalation Strategy

We encourage instructors to follow the sequence in Table 1 when assisting learners in acquiring a specific view.

**Table 1.** Graded escalation strategy for teaching POCUS image acquisition.

Step	Instructor Action	Educational Rationale
1	Allow Time	Avoid interrupting; let the learner experiment, make mistakes, and self correct. Promotes trial-and-error learning and builds confidence.
2	Identify Difficulties	Use Socratic questioning about orientation, anatomy, and image optimization. Encourages active problem-solving and self-assessment.
3	Act as a Co-Pilot	Give concise, sequential verbal instructions for probe manipulation using consistent terminology. Supports timely acquisition without removing control from the learner.
4	The Last Resort	With consent, manually guide the learner's hand, verbalizing each action. Preserves dignity and clarifies technique through kinesthetic demonstration.

This model draws inspiration from approaches in simulation-based education, where graduated assistance helps learners retain autonomy while ensuring progressive competence [3]. We train both novice and experienced instructors in this method during simulated teaching sessions and reinforce it by distributing laminated pocket cards that include the steps for teaching POCUS image acquisition (Supplementary Material - Figure S1).

## Reflections and Implications

We found that when instructors resisted the urge to “fix” the image for the learner, the learner often revealed surprising problem-solving abilities. Even if image acquisition took longer, learners retained techniques better and displayed improved orientation skills in subsequent sessions. The model also standardized instructor language and reduced variability in feedback styles.

Respecting learner autonomy in POCUS teaching aligns with broader educational principles: creating a psychologically safe space, fostering deliberate practice, and supporting the development of transferable bedside skills. While our approach was developed for undergraduate courses, it may be equally useful in postgraduate training, interprofessional education, and even continuing professional development.

## Conclusion

POCUS training should be viewed as more than a race toward producing a technically perfect image. It represents a critical opportunity to develop learners' enduring psychomotor abilities, visuospatial reasoning, and diagnostic problem-solving skills. By adopting a structured, graded escalation strategy, instructors can provide timely, targeted assistance while safeguarding learner autonomy. This approach likely supports long-term skill retention, fosters confidence, and enhances competence at the bedside.
